# Supplementary material for: Peroxisomal targeting of a protein phosphatase type 2C via mitochondrial transit
Source: Nat Commun. 2020 May 12;11:2355. doi: 10.1038/s41467-020-16146-3 (PMC7217942; doi:10.1038/s41467-020-16146-3)
Supplement: Supplementary file 1 — Supplementary Information [file 41467_2020_16146_MOESM1_ESM.pdf]

Peroxisomal targeting of a protein phosphatase type 2C via mitochondrial transit

Stehlik, et al.

Supplementary Information

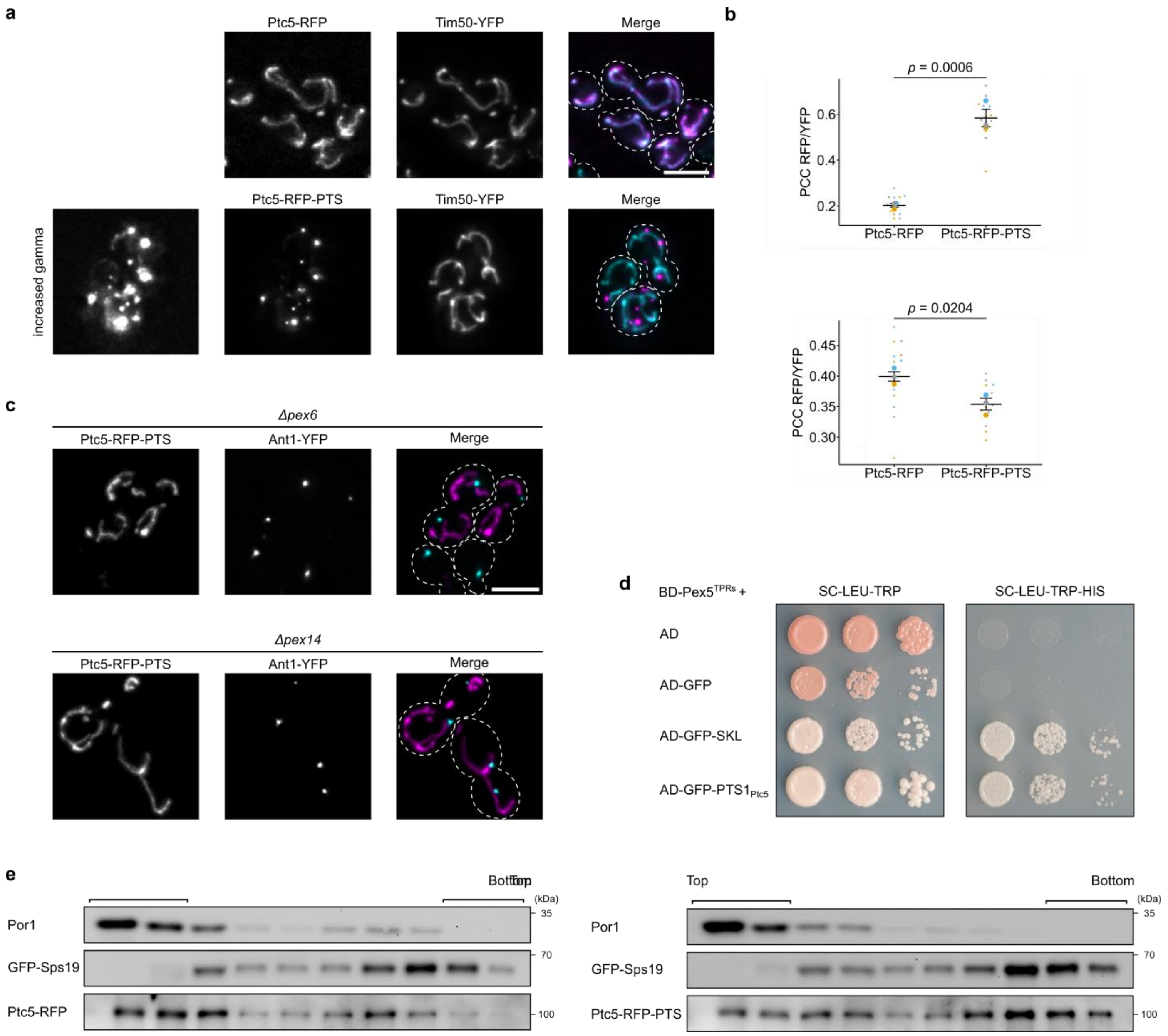

Supplementary Figure 1. Dual targeting of Ptc5-RFP-PTS to peroxisomes and to mitochondria. (**a**) Ptc5-RFP (magenta) or Ptc5-RFP-PTS (magenta) were co-expressed with the mitochondrial protein Tim50-YFP. Subcellular localization was determined by fluorescence microscopy. Image of Ptc5-RFP-PTS is also shown with increased gamma to facilitate visibility of mitochondrial localization. White color indicates colocalization. Scale bar represents 5 µm. (**b**) Correlation of Ptc5-RFP or Ptc5-RFP-PTS with either Ant1-YFP or Tim50-CFP was analyzed. PCC refers to Pearson's correlation coefficient. Quantifications are based on three independent experiment (n = 3). Each color represents one experiment. P-values were calculated with two-tailed unpaired Student's *t*-tests. (**c**) Ptc5-RFP-PTS was co-expressed with the peroxisomal protein Ant1-YFP in Δ*pex6* and Δ*pex14* mutants. Subcellular localization was determined by fluorescence microscopy. White color indicates colocalization. Scale bar represents 5 µm. (**d**) Yeast two hybrid assay. A fragment including the TPR domains of Pex5 fused to the GAL4-binding domain ($BD\text{-}Pex5^{TPRs}$) was co-expressed with the isolated Gal4-activation domain (AD), AD-GFP, AD-GFP-SKL and $AD\text{-}GFP\text{-}PTS1_{Ptc5}$ in strain AH109 Δ*pex5*, respectively. Strains were spotted in serial dilutions on SC-LEU-TRP plates to select for plasmids (growth control) and on SC-LEU-TRP-HIS plates to test for physical interaction. (**e**) Complete density gradient centrifugation analysis shown in Fig. 1e. Fractions were collected from the top of the gradient and analyzed by Western blot. Brackets specify lanes shown in Fig. 1e. Source Data are provided in the Source Data file.

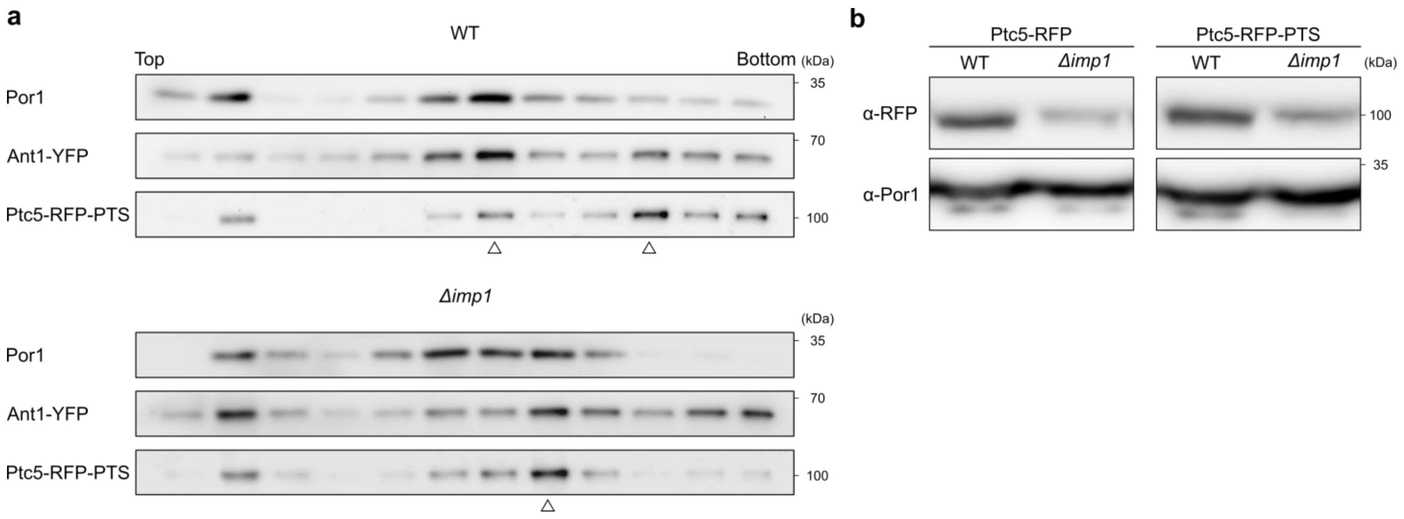

Supplementary Figure 2. Peroxisomal targeting of Ptc5-RFP-PTS depends on Imp1. (**a**) Organelles of WT and Δ*imp1* cells expressing Ptc5-RFP-PTS and Ant1-YFP grown in glucose containing medium were prepared and subjected to buoyant density centrifugation on step gradients. Fractions were collected from the top and analyzed by SDS-PAGE and Western blot. Triangles denote fractions analyzed by high resolution SDS-PAGE (Fig. 3b). (**b**) Expression of Ptc5-RFP and Ptc5-RFP-PTS was analyzed by subjecting whole cell extracts from depicted yeast strains to SDS-PAGE and Western blot. Source Data are provided in the Source Data file.

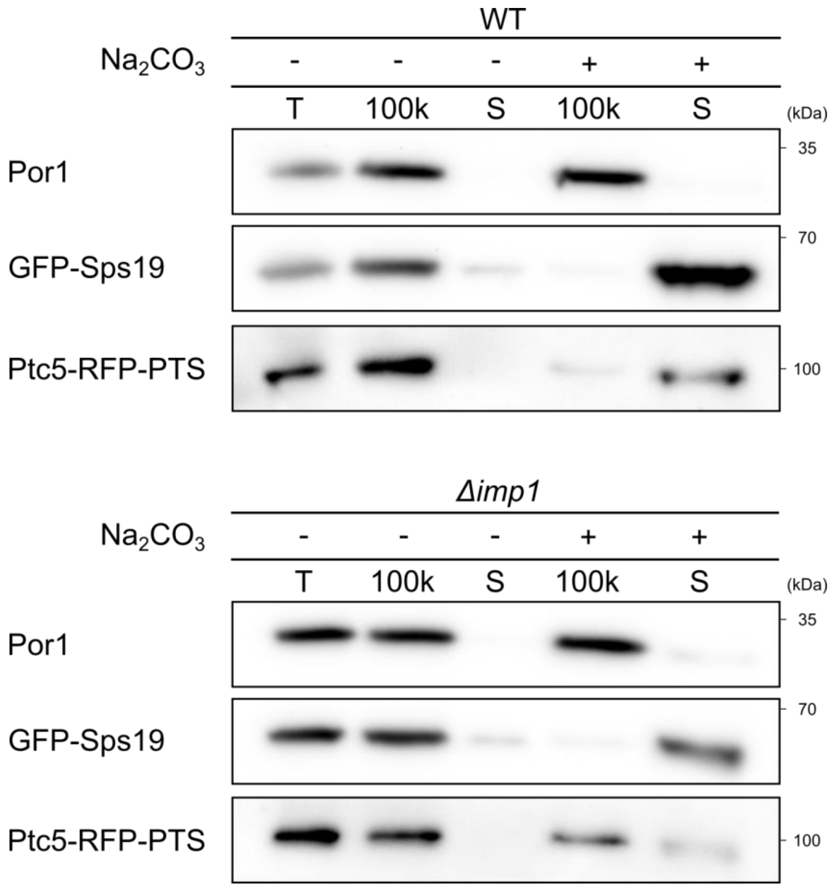

Supplementary Figure 3. Ptc5-RFP-PTS is soluble inside the peroxisomal matrix. Crude organelle preparations derived from indicated strains were subjected to Na_2CO_3 extraction and pelleted at 100.000 x g. Total (T), pellet (100k) and supernatant (S) fractions were analyzed by SDS-PAGE and Western blot. Source Data are provided in the Source Data file.

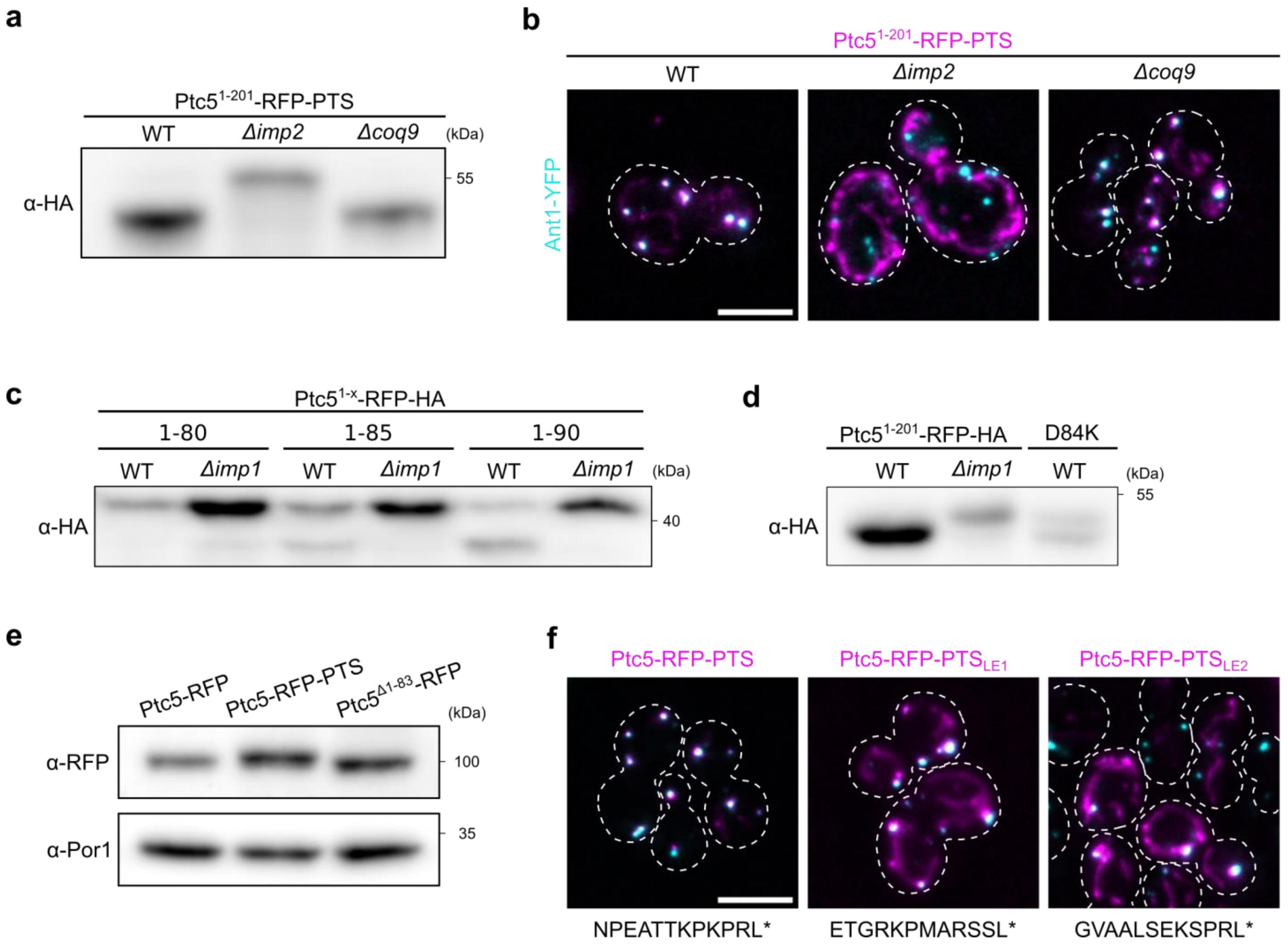

Supplementary Figure 4. Sorting to peroxisomes requires processing of Ptc5. (**a**) Whole cell lysates of indicated yeast strains were subjected to SDS-PAGE and Western blot. (**b**) Fluorescence microscopic pictures of yeast cells expressing Ptc5$^{1\text{-}201}$-RFP-PTS (magenta) and the peroxisomal membrane protein Ant1-YFP (cyan) in indicated strains. White color indicates colocalization. Scale bar represents 5 µm. (**c**) Migration of Ptc5-RFP-HA truncations from WT and Δ*imp1* cells on SDS-PAGE was determined by Western blot of whole cell lysates. (**d**) Migration of Ptc5$^{1\text{-}201}$-RFP-HA compared to Ptc5$^{1\text{-}201}$-RFP-HA containing a mutation in the Imp1 cleavage site of Ptc5 (D84K) on SDS-PAGE was examined by Western blot. (**e**) Expression of Ptc5-RFP, Ptc5-RFP-PTS and Ptc5$^{\Delta 1\text{-}83}$-RFP was analyzed by subjecting whole cell extracts to SDS-PAGE and Western blot. Por1 served as loading control. (**f**) Dual targeting of Ptc5-RFP-PTS derivatives with low efficiency (LE) PTS1 motifs was analyzed by fluorescence microscopy and compared to Ptc5-RFP-PTS (left picture). Ant1-YFP (cyan) served as marker for peroxisomes. Sequences of the C-terminal dodecamers containing PTS1 are depicted below representative pictures. White color indicates colocalization. Scale bar represents 5 µm. Source Data are provided in the Source Data file.

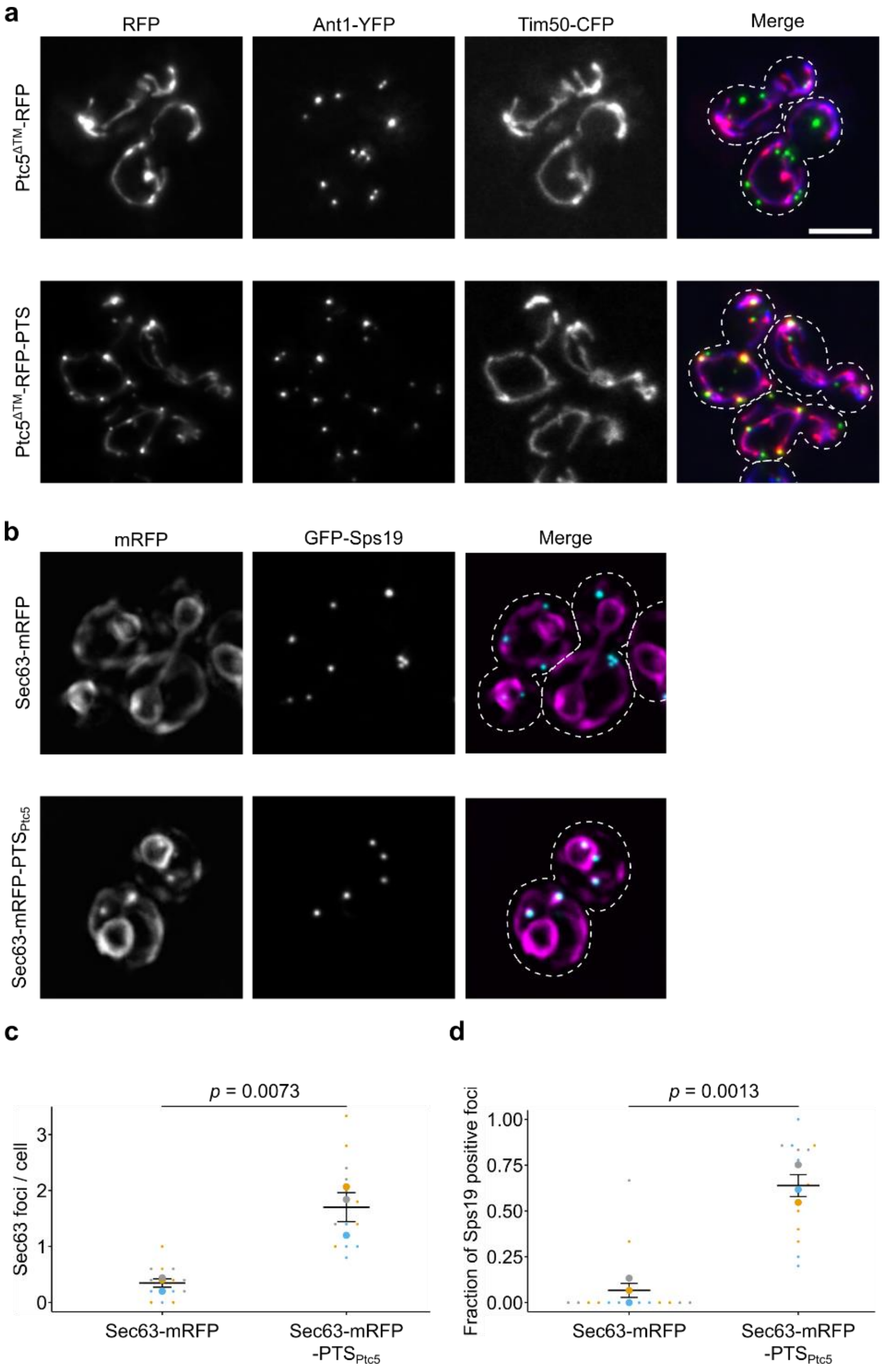

Supplementary Figure 5. Competing targeting signals increase association of organelles. (**a**) Subcellular localization of Ptc5$^{\Delta TM}$-RFP (red) and Ptc5$^{\Delta TM}$-RFP-PTS was determined using fluorescence microscopy. Ant1-YFP (green) and Tim50-CFP (blue) were used to label peroxisomes and mitochondria, respectively. Both strains were used to quantify association of peroxisomes with mitochondria. Results of this quantification are shown in Fig. 5e. Scale bar represents 5 µm. (**b**) Sec63-mRFP (magenta) and the chimeric variant Sec63-mRFP-PTS$_{Ptc5}$ (magenta) were imaged in strains containing GFP-Sps19 (cyan). White color indicates colocalization. Scale bar represents 5 µm. (**c**) The number of mRFP-positive foci per cell from three independent experiments (n = 3) was quantified. Each color represents one experiment. P-value was calculated using a two-tailed unpaired Student's *t* test. (**d**) The number of mRFP-positive foci decorated with peroxisomes from three independent experiments (n = 3) was quantified. Each color represents one experiment. P-value was calculated using a two-tailed unpaired Student's *t* test. Source Data are provided in the Source Data file.

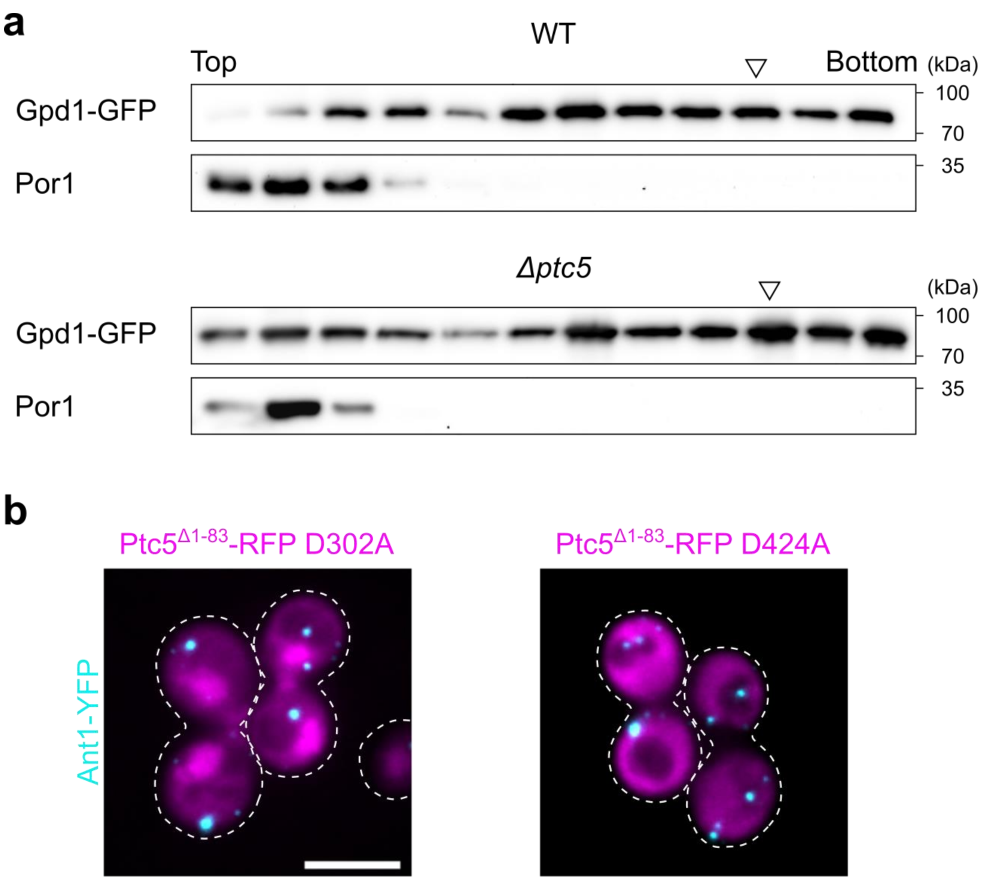

Supplementary Figure 6. Insights into the biological function of peroxisomal Ptc5 and of its unusual targeting. (**a**) Organelle preparations from indicated strains expressing GFP-Gpd1 were first pelleted at 13k x g to remove cytosolic proteins and subjected to buoyant density centrifugation on step gradients. Fractions were analyzed by SDS-PAGE and Western blot. Fractions are ordered from the lowest (Top) to the highest buoyant density (Bottom). Triangles specify fractions analyzed by PhosTag SDS-PAGE shown in Fig. 5f. (**b**) Fluorescence microscopic pictures of yeast cells expressing the enzymatic dead variants Ptc5$^{\Delta 1\text{-}83}$-RFP (D302A) or Ptc5$^{\Delta 1\text{-}83}$-RFP (D424A) (magenta) together with the peroxisomal membrane protein Ant1-YFP (cyan). White color indicates colocalization. Scale bar represents 5 µm. Source Data are provided in the Source Data file.

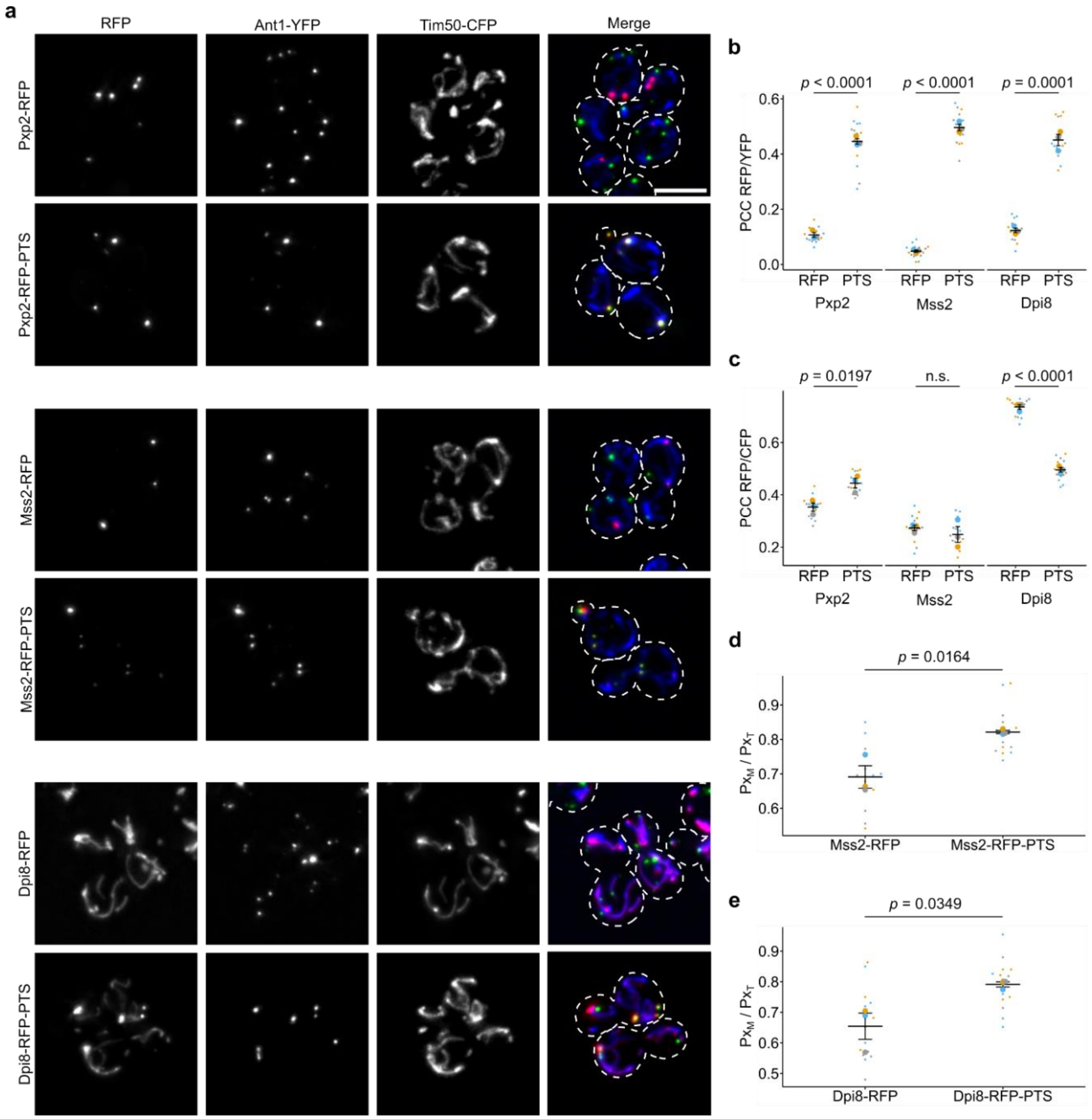

Supplementary Figure 7. Candidate mitochondrial proteins with C-terminal PTS1. (a) C-terminally or internally RFP tagged proteins (red) were imaged together with Ant1-YFP (green) and Tim50-CFP (blue). White color indicates colocalization of all three signals. Scale bar represents 5 µm. (**b and c)** Correlation of RFP with YFP or CFP signals was quantified. PCC refers to the Pearson's correlation coefficient. n.s.: not statistically significant. Quantifications from three independent experiments are shown (n = 3). Different colors represent different experiments. P-values were calculated using two-tailed unpaired Student's *t* tests. (**d**) Quantification of the fraction of peroxisomes contacting mitochondria (Px_M) in relation to the total peroxisome count (Px_T) of cells co-expressing Mss2-RFP or Mss2-RFP-PTS with Ant1-YFP and the mitochondrial membrane protein Tim50 fused to CFP. Association of Ant1-YFP containing foci with Tim50-CFP was counted. Quantifications from three independent experiments are shown (n = 3). Different s represents different experiments. P-value was calculated using a two-tailed unpaired Student's *t* test. (**e**) Quantification of the fraction of peroxisomes contacting mitochondria (Px_M) in relation to the total peroxisome count (Px_T) of cells co-expressing Dpi8-RFP or Dpi8-RFP-PTS with Ant1-YFP and the mitochondrial membrane protein Tim50 fused to CFP. Association of Ant1-YFP containing foci with Tim50-CFP was counted. Quantifications from three independent experiments are

shown (n = 3). Colors represent different experiments. P-value was calculated using a two-tailed unpaired Student's *t* test. Source Data are provided in the Source Data file. Source Data are provided in the Source Data file.

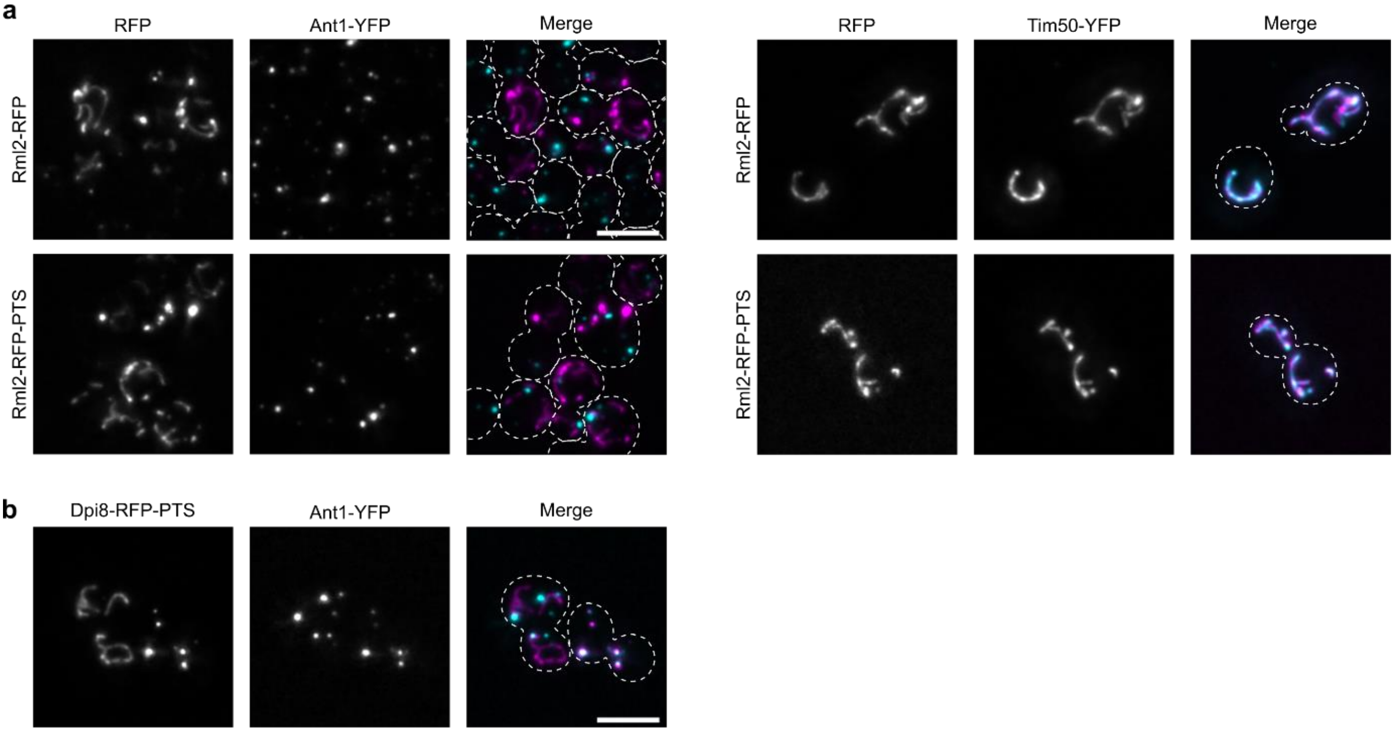

Supplementary Figure 8. Candidate mitochondrial proteins with C-terminal PTS1. (**a**) C-terminally (upper panel) or internally (lower panel) RFP tagged Rml2 (magenta) was imaged together with Ant1-YFP (cyan, left panel) or Tim50-YFP (cyan, right panel). White color indicates colocalization. Scale bar represents 5 µm. (**b**) Internally RFP tagged Dpi8 (magenta) was imaged together with Ant1-YFP (cyan). Note that Dpi8-RFP-PTS is localized almost entirely to mitochondria in one depicted cell, while it predominantly localizes to peroxisomes in the other cell. This indicates regulation of tug-of-war like sorting.

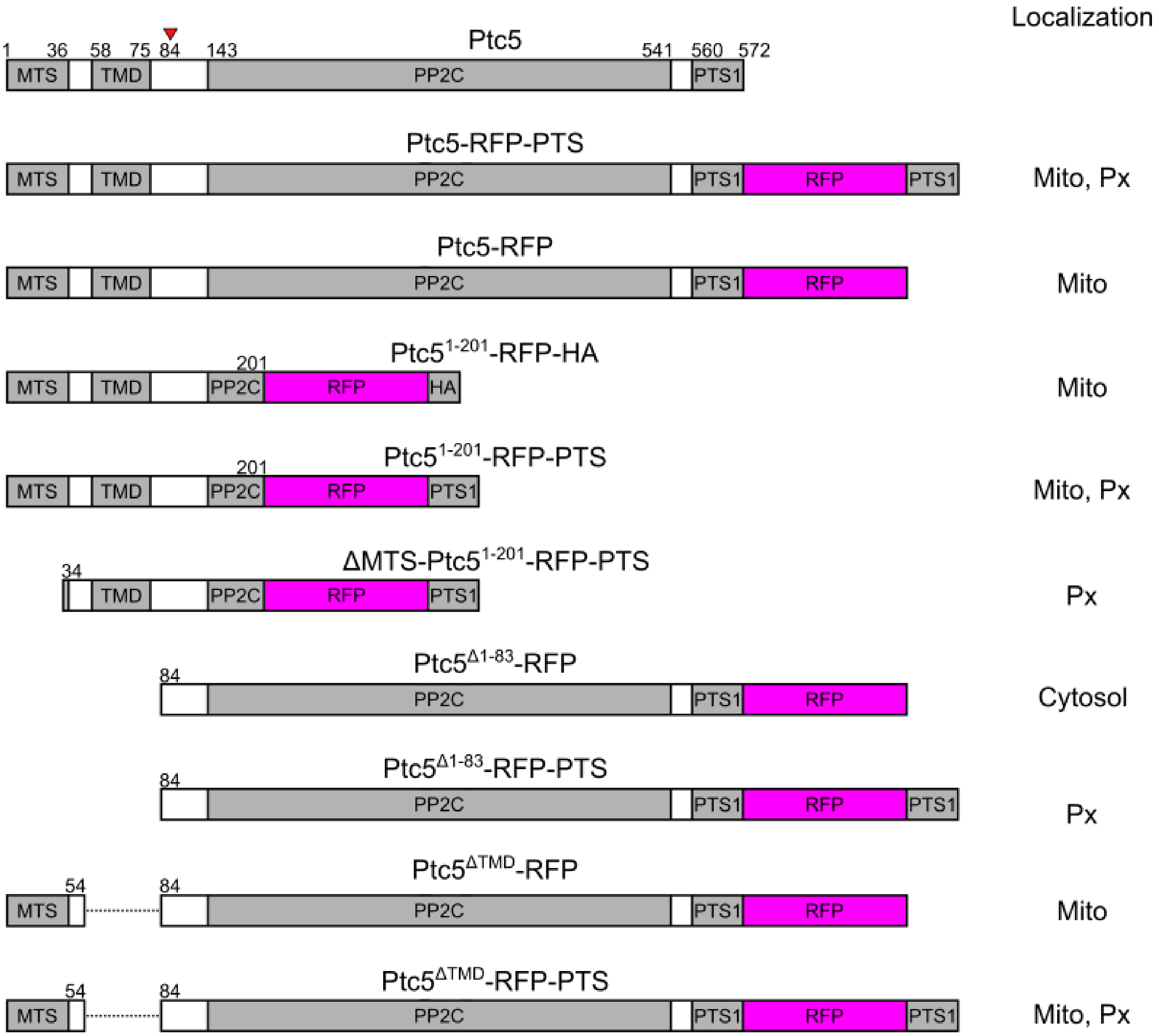

Supplementary Figure 9. Schematic representation of Ptc5-RFP fusion constructs generated during this study. The red triangle indicated the Imp cleavage site. The subcellular localization of respective fusion proteins is highlighted.

Supplementary Table 1. Mitochondrial proteins with PTS1.

Standard name	Systematic name[1]	Putative PTS1[2]	P-Score[3]	Prediction[4]
CAT2	YML042W	ALENENKRKAKL	11.229	Targeted
PTC5	YOR090C	NPEATTKPKPRL	10.411	Targeted
MRP7	YNL005C	IARSRRAFLSKL	9.854	Targeted
TES1	YJR019C	VYGSERDIRAKF	9.571	Targeted
MSS2	YDL107W	KDSIKLLDKARL	9.328	Targeted
CIT2	YCR005C	YKELVKNIESKL	9.312	Targeted
PET309	YLR067C	RKSKRVLPVSKF	8.830	Targeted
LYS4	YDR234W	KGGLEGWVKSQL	8.383	Targeted
NSA1	YGL111W	VAASKASKKSKI	7.400	Targeted
MIC10	YCL057C-A	FRSSAGLRSSKV	7.189	Targeted
LYS12	YIL094C	TQQVVDDVLSRL	7.008	Targeted
DPI8	YJL133C-A	ATRRHLAHAPKL	6.028	Targeted
CTA1	YDR256C	KHASELSSNSKF	4.375	Targeted
MRS1	YIR021W	AGSSKFLKGAKI	2.750	Targeted
MRPL37	YBR268W	QRIKQNNFLSQL	1.700	Targeted
UTP6	YDR449C	RYKILDLIISKL	0.611	Targeted
RML2	YEL050C	VKDRPRGKDARL	0.073	Targeted
PXP2	YJR111C	CGVSWKSGVVKL	-1.169	Twilight zone
ATP8	Q0080	RLYVSRLFISKL	-5.777	Twilight zone
SOD2	YHR008C	KEASRRFDAGKI	-8.224	Twilight zone
MIN10	YFR032C-B	PISPIGNAGSQI	-10.522	Not targeted
DSS1	YMR287C	DCLEGMLELEKL	-16.365	Not targeted
NDE1	YMR145C	AKVYFLGRDSSI	-17.919	Not targeted
TMA19	YKL056C	AIWKHGIVEEKI	-20.006	Not targeted
EXG2	YDR261C	LAITIAALCASL	-24.671	Not targeted
CAT5	YOR125C	ICRVAIWSAERI	-32.755	Not targeted

[1]: Each yeast ORF is assigned a systematic name in the form of a seven-character alphanumeric formula. The first three letters define the host origin (Y, for yeast), the specific chromosome and whether the ORF lies to the left (L) or right (R) of the centromere. This is followed by three digits listing the relative ORF position from the centromere. Finally, the letter W or C is inserted to indicate expression from the Watson or Crick strand of DNA.

[2]: Dodecamer at the extreme C-Terminus of the given protein.

[3] and [4]: The P-Score represents the bioinformatically calculated probability that the given protein sequence contains a functional PTS1. Peptides with a score higher than 0 are predicted to be **targeted** to peroxisomes, peptides with a score lower than -10 are predicted to be **not targeted**. Peptides with a score between -10 and 0 can't be predicted reliably (**Twilight zone**).

Supplementary Table 2. Yeast strains, plasmids and oligonucleotides.

Yeast strains

Name	Genotype	Reference
BY4741	*MATa his3Δ1 leu2Δ0 met15Δ0 ura3Δ0*	Brachmann *et al* (1998)
AH109	*MATa, trp1-901, leu2-3, 112, ura3-52, his3-200, gal4Δ, gal80Δ,* LYS2::$GAL1_{UAS}$-$GAL1_{TATA}$-HIS3, $GAL2_{UAS}$-$GAL2_{TATA}$-ADE2, URA3::$MEL1_{UAS}$-$MEL1_{TATA}$-lacZ	Clontech
YTS398	*AH109 pex5::NAT*	this study
YTS72	NAT::P_{ADH}-gfp-sps19	this study
YTS123	*tim50-yfp::NAT*	this study
YTS129	*ant1-yfp::NAT*	this study
Y11866	*ptc5::kanMX4*	Euroscarf
Y03603	*pex5::kanMX4*	Euroscarf
Y11115	*pex6::kanMX4*	Euroscarf
Y14520	*pex14::kanMX4*	Euroscarf
Y10732	*imp1::kanMX4*	Euroscarf
Y10611	*imp2::kanMX4*	Euroscarf
Y14150	*coq9::kanMX4*	Euroscarf
YTS109	Y03603 *ant1-yfp::NAT*	this study
YTS121	Y10732 *ant1-yfp::NAT*	this study
YTS303	Y11115 *ant1-yfp::NAT*	this study
YTS302	Y14520 *ant1-yfp::NAT*	this study
YTS192	Y10611 *ant1-yfp::NAT*	this study
YTS193	Y14150 *ant1-yfp::NAT*	this study
YTS191	Y10732 NAT::P_{ADH}-gfp-sps19	this study
YTS218	*ant1-yfp::NAT ptc5-3ha::kanMX4*	this study
YTS221	*ant1-yfp::NAT ptc5-3ha-pts::kanMX4*	this study
YTS402	*ant1-yfp::NAT ptc5-3myc-pts::kanMX4*	this study
YTS404	*ant1-yfp::NAT ptc5-3myc::kanMX4*	this study
YTS406	*ant1-yfp::NAT imp2-3myc::kanMX4*	this study
YTS411	NAT::P_{ADH}-gfp-sps19 ptc5-3ha-pts::kanMX4	this study
YTS413	NAT::P_{ADH}-gfp-sps19 ptc5-3ha-pts::kanMX4	this study
YTS185	*gpd1-gfp::hph*	this study
YTS183	Y11866 *gpd1-gfp::hph*	this study
YTS145	NAT::P_{ADH}-gfp-sps19 P_{SEC63}-sec63-mrfp-ura3::leu2	this study
YTS146	NAT::P_{ADH}-gfp-sps19 P_{SEC63}-sec63-mrfp-pts_{PTC5}-ura3::leu2	this study
YTS187	*ant1-yfp::NAT tim50-cfp::kanMX4*	this study

Plasmids

Name	Genotype	Reference
pCT310	HIS3 ARS/CEN P_{GAL1}-YFP-$p14^{D122Y}$-$TEV^{234STOP}$	Renicke *et al* (2013)
pGBKT7	TRP1 2 µ P_{ADH1}-GAL4-BD	Clontech
pGADT7	LEU2 2 µ P_{ADH1}-NLS_{SV40}-GAL4-AD	Clontech
pTS72	P_{TEF1}-PTC5-RFP-PTS in pCT310	this study
pTS73	P_{TEF1}-PTC5-RFP in pCT310	this study
pTS97	P_{TEF1}-$PTC5^{1\text{-}201}$-RFP-HA in pCT310	this study
pTS98	P_{TEF1}-$PTC5^{1\text{-}201}$-RFP-PTS in pCT310	this study
pTS160	P_{TEF1}-$PTC5^{1\text{-}201(D84K)}$-RFP-HA in pCT310	this study
pTS161	P_{TEF1}-$PTC5^{1\text{-}201(\Delta 81\text{-}84)}$ -RFP-HA in pCT310	this study
pTS162	P_{TEF1}-$PTC5^{\Delta 81\text{-}84}$-RFP-PTS in pCT310	this study
pTS151	P_{TEF1}-$PTC5^{1\text{-}80}$-RFP-HA in pCT310	this study
pTS152	P_{TEF1}-$PTC5^{1\text{-}85}$-RFP-HA in pCT310	this study
pTS153	P_{TEF1}-$PTC5^{1\text{-}90}$-RFP-HA in pCT310	this study
pTS275	P_{TEF1}-$PTC5^{\Delta 1\text{-}83}$-RFP in pCT310	this study
pTS252	P_{TEF1}-$PTC5^{\Delta 1\text{-}83}$-RFP-PTS in pCT310	this study
pTS305	P_{TEF1}-$PTC5^{\Delta 1\text{-}83(D302A)}$-RFP in pCT310	this study
pTS306	P_{TEF1}-$PTC5^{\Delta 1\text{-}83(D424A)}$-RFP in pCT310	this study
pTS288	P_{TEF1}-$PTC5^{\Delta TM}$-RFP in pCT310	this study
pTS289	P_{TEF1}-$PTC5^{\Delta TM}$-RFP-PTS in pCT310	this study
pTS297	P_{TEF1}-PTC5-RFP-PTS_{LE1} in pCT310	this study
pTS298	P_{TEF1}-PTC5-RFP-PTS_{LE2} in pCT310	this study
pSM1960	URA3 2µ P_{SEC63}-SEC63-RFP	Metzger *et al* (2008)
pTS148	URA3 2µ P_{SEC63}-SEC63-RFP-PTS_{PTC5}	this study
pTS295	P_{TEF1}-PXP2-RFP in pCT310	this study
pTS296	P_{TEF1}-PXP2-RFP-PTS in pCT310	this study
pTS309	P_{TEF1}-MSS2-RFP in pCT310	this study
pTS310	P_{TEF1}-MSS2-RFP-PTS in pCT310	this study

Name	Genotype	Reference
pTS311	*P_{TEF1}-RML2-RFP in pCT310*	this study
pTS312	*P_{TEF1}-RML2-RFP-PTS in pCT310*	this study
pTS313	*P_{TEF1}-DPI8-RFP in pCT310*	this study
pTS314	*P_{TEF1}-DPI8-RFP-PTS in pCT310*	this study

Oligonucleotides

Name	Sequence
MI801	tgctacttcaatagttatgaacccggaggcaacaacaaagccaaaatgaacttctaaataagcgaatttc
MI802	tataaaataaatcctctggtatatacctacctcagcataagtttatatcgatcgatgaattcgagctcg
MJ484	atgattacgccaagcgcgcaattaaccctcactaaagggaacaaaagctggatcccccacacaccatagc
MJ485	cttaaccgttttctttatagctacggttctagttaagggagacatggatcctttgtaattaaaacttagattagattgc
MJ221	ttcatatgcatattttctttaattaattcagacattaatctaggttttggctttg
MJ222	acccggaggcaacaacaaagccaaaacctagattaatgtctgaattaattaaag
MJ223	atgtaagcgtgacataactaattacatgactcgagttatttatgacctaatttag
MJ224	atgtaagcgtgacataactaattacatgactcgagtcataatctaggttttggctttgttgttgcctccgggtttttatgacctaatttagatg
MK223	cgaaaaaagtgggggaaagtatgatatgttatctttctccaataaatctaatcgatgaattcgagctcg
MK224	caatgaagaacctgccggacatgattgaagaattagatctacatgaagatcgtacgctgcaggtcgac
MK272	ttacgccaagcgcgcaattaaccctcactaaagggaacaaaagctgatcccccacacaccatagc
MK230	tcctatttttggatatatatacatcaataaacaatatatcataacacatgcgtacgctgcaggtcgac
MK231	atgaatttgggcagtgatgcgagaacataaaattgcggagaaccatatcaatcgatgaattcgagctcg
MK365	Atatgaaaacgttcttcggggcg
MK366	atatggcgcgcctcataacctaggctttggttttgtggttgcttccggattgcactgagcagcgtaatctg
MK386	agcatagcaatctaatctaagttttaattacaaaggatccatgtctcccttaactagaaccg
MK387	ataatttcatatgcatattttctttaattaattcagacataccaccatgtccatcaaaaattc
MK388	Atgtctgaattaattaaagaaaatatgcatatg
MK389	aatgtaagcgtgacataactaattacatgactcgagtcacgcatagtcaggaacatcgtatgggtaaaagattcctttatgacctaatttagatggtaaatcac
MK499	taagcgtgacataactaattacatgactcgagtcataatctaggttttggctttgttgttgcctccgggttcgcatagtcaggaacatcg
MK500	catagcaatctaatctaagttttaattacaaaggatccatggatacaattaacggaataaaagattattc
MK514	Acaattaacggaataaaagattattctac
MK515	tagcaatctaatctaagttttaattacaaaggatccatgtctcccttaactagaacc
MK529	atgcatattttctttaattaattcagacatgatctttttaggggaatagtaggag
MK528	tgtagaataatcttttattccgttaattgttttaagggagaggatctttttaggggaatag
MK530	atgcatattttctttaattaattcagacattgtatcaagggagaggatctttttag
MK531	atgcatattttctttaattaattcagacatttttattccgttaattgtatcaaggg
MK564	Tccgttaattgtgatctttttaggggaatagtaggag
MK565	Ccctaaaaagatcacaattaacggaataaaagattattctac
MK777	Aatcttttattccgttaattgtatctgatgatatgaatagctttgtccttg
MK778	Gatacaattaacggaataaaagattattctac
MK834	gggagggcgtgaatgtaagcgtgacataactaattacatgactcgagttacaaggagctcctggccattggcttccgacctgtttctttatgacctaatttagatgg
MK835	gggagggcgtgaatgtaagcgtgacataactaattacatgactcgagtcagagacgtggcgacttttccgagagagcagcgacacctttatgacctaatttagatgg
MK860	Aaagcccttgaggctccagtgacagccactttc
MK861	Gctgtcactggagcctcaagggctttaatttgtgg
MK862	Ttcgaataaaccggccgaacccatcaccataaatttag
MK863	Gatgggttcggccggtttattcgaattactaactaatgaag
MK864	atctaatctaagttttaattacaaaggatccatgaaccagatactaaatgcac
MK865	Taattcagacatcaattttacaacaccagatttcc
MK866	Tgttgtaaaattgatgtctgaattaattaaagaaaatatgc
MK867	aagcgtgacataactaattacatgactcgagttacaattttacaacaccagatttccaactaacaccacatttatgacctaatttagatggtaaatc
MK870	atctaatctaagttttaattacaaaggatccatgcagaggtttgtcagtaag
MK871	Ttaattcagacataagccgtgctttgtccagc
MK872	Aaagcacggcttatgtctgaattaattaaagaaaatatgc
MK873	aagcgtgacataactaattacatgactcgagttaaagccgtgctttgtccagcaactttatggaatctttttatgacctaatttagatggtaaatc
MK874	Atctaatctaagttttaattacaaaggatccatgttggtgctagggtctc
MK875	Ttaattcagacattaatcttgcatctttgcctc
MK876	Gatgcaagattaatgtctgaattaattaaagaaaatatgc
MK877	aagcgtgacataactaattacatgactcgagttataatcttgcatctttgcctcttggtctgtcttttactttatgacctaatttagatggtaaatc
MK878	atctaatctaagttttaattacaaaggatccatgatcgcccaaagtaccag
MK879	Ttaattcagacatcaactttggcgcgtgagc
MK880	Gcgccaaagttgatgtctgaattaattaaagaaaatatgc
MJ789	acgccttatttgaagaggaaaagaaaaagaagaagattgctgaatccaaacgtacgctgcaggtcgac
MJ790	tacacacatagatacgtagatacatgagaagagggtttacatgaaaattaatcgatgaattcgagctcg
MK881	aagcgtgacataactaattacatgactcgagctacaactttggcgcgtgagccaagtgtcttctggtagctttatgacctaatttagatggtaaatc
ML242	atatggcgcgcctcataacctaggctttggttttgtggttgcttccggattgactctagatgatccgttcaagtc
ML246	tctaatctaagttttaattacaaaggatccatggcatattcaacatcacatgcc
ML295	Tattttctttaattaattcagacatgtaccatatattcttcagtttagggtg
ML296	Tattttctttaattaattcagacattagcatggcatagtggtataatttagg
Ptc5_ko_for	atgtctcccttaactagaaccgtagctataaagaaaacggttaagcgtacgctgcaggtcgac
Ptc5_ko_rev	aaatcctctggtatatacctacctcagcataagtttatatcgtcaatcgatgaattcgagctcg

**Name**	**Sequence**
Sps19_S1	acacttttagagtttgggaggtcgtatgttcgagtcatggatactcgtacgctgcaggtcgac
Sps19_S4	agaaccctcagtaacgaatttgccgtccaaagtgtttgctgtattcatcgatgaattctctgtcg
Ant1_S3	tctttttcctaaagcacaacggacaacgcaagctggcttccactcgtacgctgcaggtcgac
Ant1_S2	tgagaatccttactattactgaaataagcacattgcgtttagaatatcgatgaattcgagctcg
Pex5-TPR_for	Atatgaattctctaacaacgaatattttaataatcctaatgc
Pex5-TPR_rev	Atatggatcctcaaaacgaaaattctcctttaaatcttttcagg
Gfp-2H_for	Atatgaattcggagcaggtgctggtgctg
Gfp-2H_rev	Atatggatccttatttgtacaattcatccataccatgg
GFP_Ptc5-_2H_rev	atatggatccttataatctaggttttggctttgttgttgcctccgggtttttgtacaattcatccataccatgg
GFP_SKL-2H_rev	Atatggatccttaaagcttacttttgtacaattcatccataccatgg

1. Brachmann, C. B. *et al.* Designer deletion strains derived from Saccharomyces cerevisiae S288C: a useful set of strains and plasmids for PCR-mediated gene disruption and other applications. *Yeast* **14**, 115–132 (1998).

2. Renicke, C., Spadaccini, R. & Taxis, C. A Tobacco Etch Virus Protease with Increased Substrate Tolerance at the P1' position. *PLoS One* **8**, e67915 (2013).

3. Metzger, M. B., Maurer, M. J., Dancy, B. M. & Michaelis, S. Degradation of a cytosolic protein requires endoplasmic reticulum-associated degradation machinery. *J. Biol. Chem.* **283**, 32302-32316 (2008).
