# Supplementary material for: Peroxisomal targeting of a protein phosphatase type 2C via mitochondrial transit
Source: Nat Commun. 2020 May 12;11:2355. doi: 10.1038/s41467-020-16146-3 (PMC7217942; doi:10.1038/s41467-020-16146-3)
Supplement: Supplementary file 3 — Description of Additional Supplementary Files [file 41467_2020_16146_MOESM3_ESM.pdf]

Description of Additional Supplementary Files

File Name: Supplementary Data 1
Description: Proteomics dataset. Available on PRIDE under accession number PXD018591.
