# Supplementary material for: Peroxisomal targeting of a protein phosphatase type 2C via mitochondrial transit
Source: Nat Commun. 2020 May 12;11:2355. doi: 10.1038/s41467-020-16146-3 (PMC7217942; doi:10.1038/s41467-020-16146-3)
Supplement: Supplementary file 5 — Reporting Summary [file 41467_2020_16146_MOESM5_ESM.pdf]

nature research

Corresponding author(s): Johannes Freitag

Last updated by author(s): Mar 30, 2020

Reporting Summary

Nature Research wishes to improve the reproducibility of the work that we publish. This form provides structure for consistency and transparency in reporting. For further information on Nature Research policies, see Authors & Referees and the Editorial Policy Checklist .

Statistics

For all statistical analyses, confirm that the following items are present in the figure legend, table legend, main text, or Methods section.

n/a	Confirmed	
☐	☒	The exact sample size (*n*) for each experimental group/condition, given as a discrete number and unit of measurement
☐	☒	A statement on whether measurements were taken from distinct samples or whether the same sample was measured repeatedly
☐	☒	The statistical test(s) used AND whether they are one- or two-sided<br>*Only common tests should be described solely by name; describe more complex techniques in the Methods section.*
☒	☐	A description of all covariates tested
☒	☐	A description of any assumptions or corrections, such as tests of normality and adjustment for multiple comparisons
☐	☒	A full description of the statistical parameters including central tendency (e.g. means) or other basic estimates (e.g. regression coefficient) AND variation (e.g. standard deviation) or associated estimates of uncertainty (e.g. confidence intervals)
☐	☒	For null hypothesis testing, the test statistic (e.g. *F*, *t*, *r*) with confidence intervals, effect sizes, degrees of freedom and *P* value noted<br>*Give P values as exact values whenever suitable.*
☒	☐	For Bayesian analysis, information on the choice of priors and Markov chain Monte Carlo settings
☒	☐	For hierarchical and complex designs, identification of the appropriate level for tests and full reporting of outcomes
☐	☒	Estimates of effect sizes (e.g. Cohen's *d*, Pearson's *r*), indicating how they were calculated

Our web collection on statistics for biologists contains articles on many of the points above.

Software and code

Policy information about availability of computer code

Data collection	Image aquisition was performed using Volocity 5.5.2.<br>Chemiluminescence was recorded using ChemoStar Imager
Data analysis	Plots were generated using RStudio 1.2.1335 with R 3.6.0.<br>Image analysis and deconvolution was performed using ImageJ 1.52a.<br>Data was arranged and stored using Microsoft Excel 2016<br>MS/MS data analysis was performed using Mascot embedded in Proteom Discover 1.4.<br>FASTA files were analyzed using notepad++ 7.8.4.<br>Prediction of PTS1 was performed using PTS-Predictor (http://mendel.imp.ac.at/pts1/), Neuberger et al (2003).

For manuscripts utilizing custom algorithms or software that are central to the research but not yet described in published literature, software must be made available to editors/reviewers. We strongly encourage code deposition in a community repository (e.g. GitHub). See the Nature Research guidelines for submitting code & software for further information.

Data

Policy information about availability of data

All manuscripts must include a data availability statement. This statement should provide the following information, where applicable:

- Accession codes, unique identifiers, or web links for publicly available datasets
- A list of figures that have associated raw data
- A description of any restrictions on data availability

Non-cropped western blots and data underlying all plots are provided as Source Data file. Any other original data to support findings of this study are available from the authors on request.

Field-specific reporting

Please select the one below that is the best fit for your research. If you are not sure, read the appropriate sections before making your selection.

- [x] Life sciences
- [ ] Behavioural & social sciences
- [ ] Ecological, evolutionary & environmental sciences

For a reference copy of the document with all sections, see nature.com/documents/nr-reporting-summary-flat.pdf

Life sciences study design

All studies must disclose on these points even when the disclosure is negative.

Sample size

Microscopic data was collected from three independent S. cerevisiae cultures. Five images per culture were quantified. Plots are described in more detail in the methods section. P-values were calculated using an two-tailed unpaired Student's t-test. Pearson's correlation coefficient was calculated with Volocity 5.5.2. For calculation of pearson's correlation coefficients all analyzed images contained ten or more cells and one image represents one data point. For quantification of contacts between mitochondria and peroxisomes 5 cells per image per strain from three independent experiments were analyzed. Quantification was performed by manual inspection of cells showing a signal in the RFP channel. Inspection was carried out without knowledge of the respective genotypes. Sample size was not predetermined. We always made efforts to investigate a large number of cells, which is possible for S. cerevisiae.

Data exclusions

No data were excluded.

Replication

Unless stated otherwise, all experiments were repeated at least three times. All attempts were sucessful.

Randomization

Yeast strains were imaged in random order for all datasets.

Blinding

Quantification of microscopic datasets was performed without knowledge of the respective genotypes of the imaged strains.

Reporting for specific materials, systems and methods

We require information from authors about some types of materials, experimental systems and methods used in many studies. Here, indicate whether each material, system or method listed is relevant to your study. If you are not sure if a list item applies to your research, read the appropriate section before selecting a response.

Materials & experimental systems

n/a	Involved in the study	
☐	☒	Antibodies
☒	☐	Eukaryotic cell lines
☒	☐	Palaeontology
☒	☐	Animals and other organisms
☒	☐	Human research participants
☒	☐	Clinical data

Methods

n/a	Involved in the study	
☒	☐	ChIP-seq
☒	☐	Flow cytometry
☒	☐	MRI-based neuroimaging

Antibodies

Antibodies used

Primary antibodies:
anti-GFP (1:5.000 (WB), 1:500 (IF), TP401, Torrey Pines Biolabs)
anti-HA (1:2.500 (WB), ab1302275, Abcam)
anti-tagRFP (1:1.000 (WB), AB233, Evrogen)
anti-Myc (1:1000 (WB), 1:250 (IF), #2276, Cell Signaling Technology)
rabbit anti-Por1 (1:1000 (WB), provided by Prof. Roland Lill (University of Marburg)
Secondary antibodies:
m-IgGκBP-HRP (1:5.000, sc-516102, Santa Cruz Biotechnology)
mouse anti-rabbit IgG-HRP (1:5.000, sc-2357, Santa Cruz Biotechnology)
Alexa Fluor 488 AffiniPure Donkey anti-rabbit (1:200, 711-545-152, Jackson ImmunoResearch)
Alexa Fluor 594 AffiniPure Donkey anti-mouse (1:200, 715-585-150, Jackson ImmunoResearch)

Validation

Primary antibodies:
anti-GFP (1:5.000 (WB), 1:500 (IF), TP401, Torrey Pines Biolabs), Manufacturer's data sheet.
anti-HA (1:2.500 (WB), ab1302275, Abcam), Manufacturer's data sheet.
anti-tagRFP (1:1.000 (WB), AB233, Evrogen), Manufacturer's data sheet.
anti-Myc (1:1000 (WB), 1:250 (IF), #2276, Cell Signaling Technology), Manufacturer's data sheet.

rabbit anti-Por1 (1:1000 (WB), provided by Prof. Roland Lill (University of Marburg), Künkele et al., 1998; works similar as anti-VDAC1/Por1 antibody (ab110326, Abcam)
