# Supplementary material for: Peroxisomal targeting of a protein phosphatase type 2C via mitochondrial transit
Source: Nat Commun. 2020 May 12;11:2355. doi: 10.1038/s41467-020-16146-3 (PMC7217942; doi:10.1038/s41467-020-16146-3)
Supplement: Supplementary file 6 — Source Data [file 41467_2020_16146_MOESM6_ESM.zip › Western Blots.docx]

**Western Blots**

Fig. 1e / Fig. S1

Ptc5-RFP

kDa

kDa

Ptc5-RFP-PTS

170


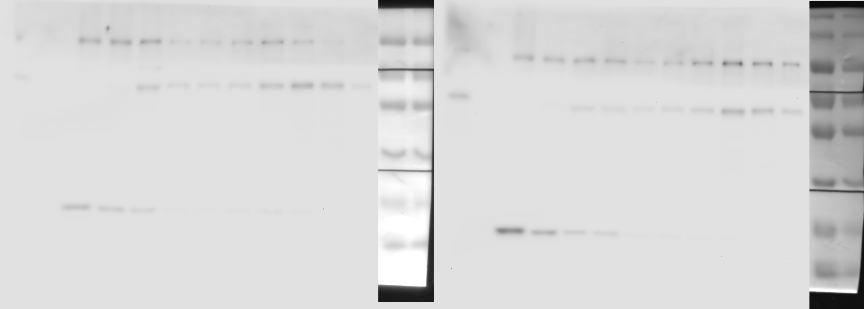


Anti-Por1

Anti-GFP

25

35

40

55

70

100

130

25

35

40

55

70

100

Fig. 2c

Ptc5-3xMyc

Ptc5-3xMyc-PTS

kDa

kDa


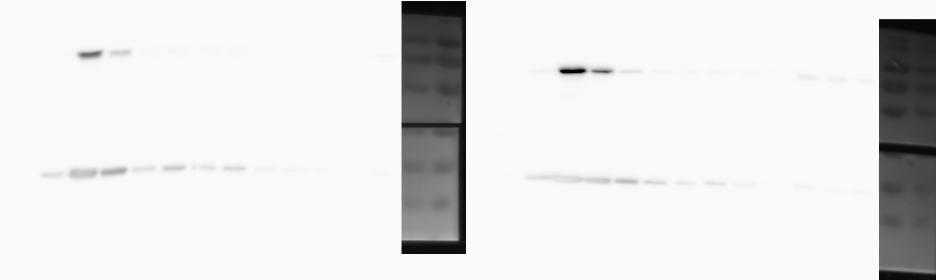

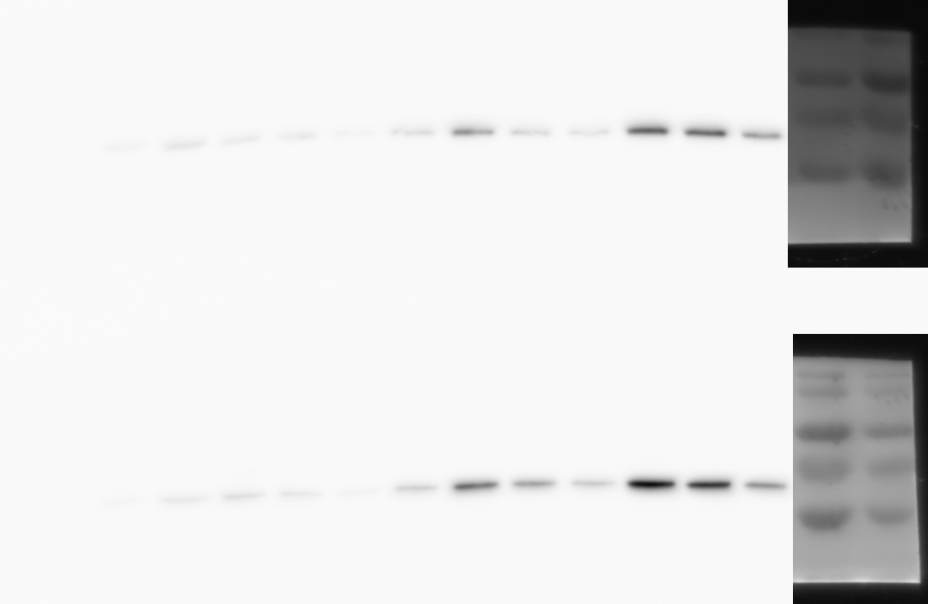


Anti-GFP

Anti-GFP

Ptc5-3xMyc-PTS

Ptc5-3xMyc

Anti-Myc

Anti-Por1

170

130

100

55

70

kDa

70

55

100

130

kDa

25

170

130

70

100

35

40

55

100

70

55

40

35

25

Fig. 3c


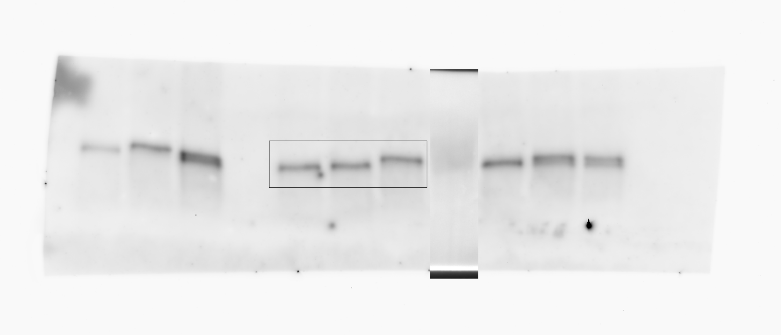


Anti-tagRFP

100 kDa

Fig. 3d


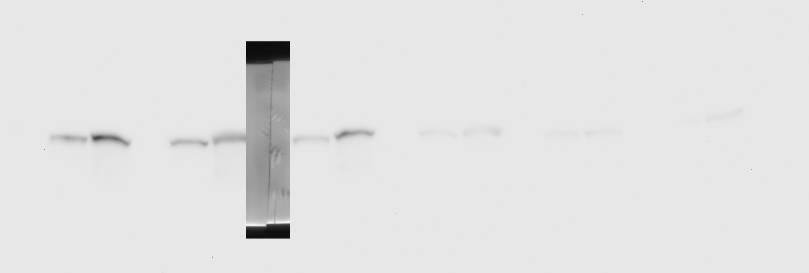


Anti-Myc

100 kDa

70 kDa

Fig. 3e


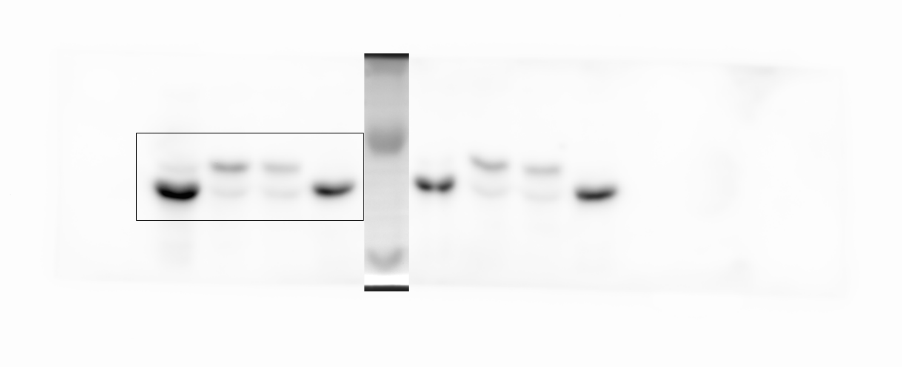


Anti-HA

40 kDa

55 kDa

Fig. 3g


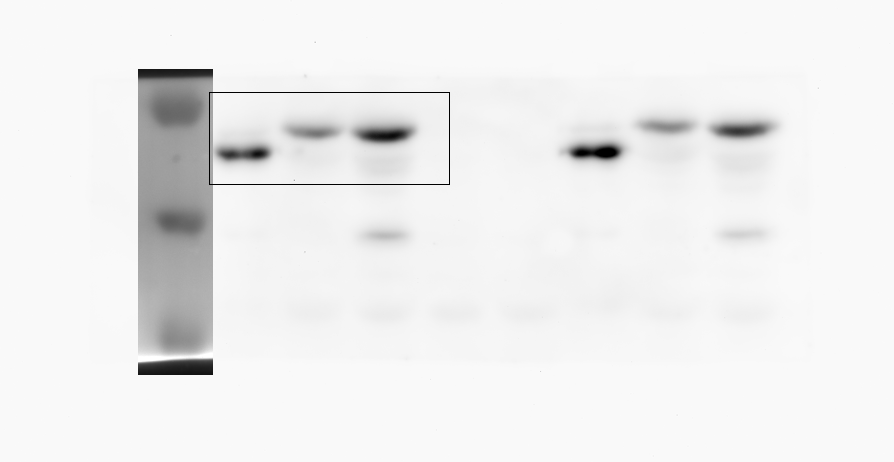


Anti-HA

55 kDa

40 kDa

Fig. 4a

kDa


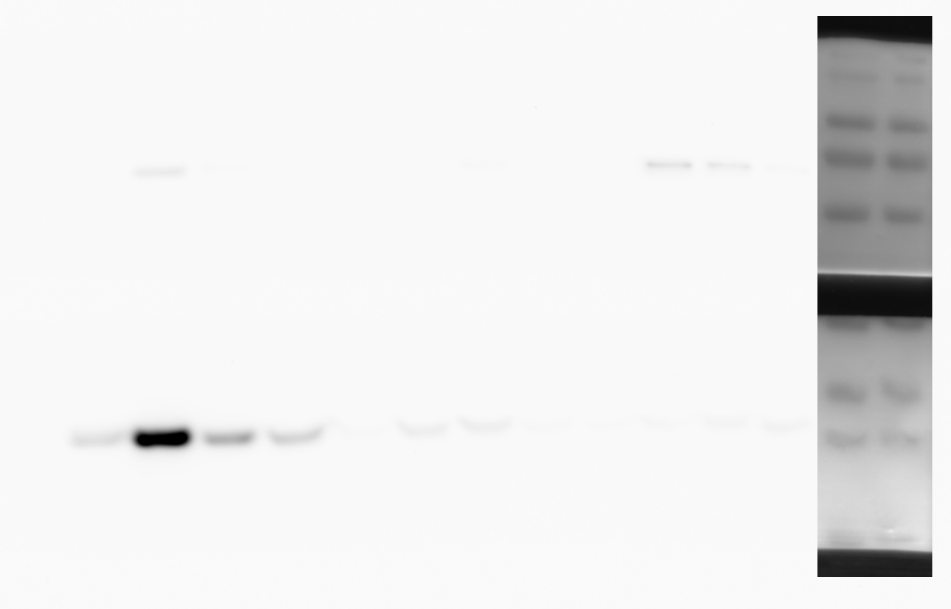


Anti-Myc

40

kDa

170

130

100

70

55

40

35

25

Anti-GFP


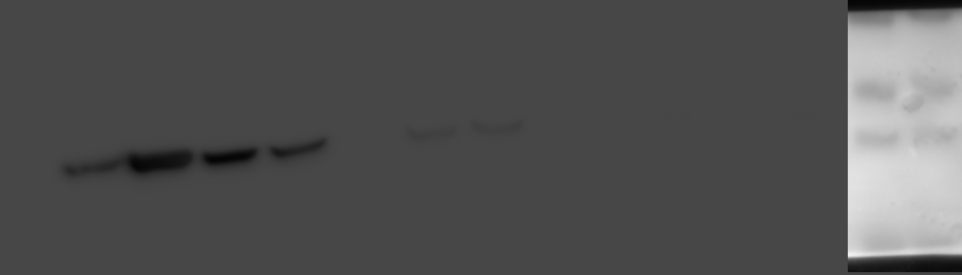


25

35

Anti-Por1

Fig. 4b

kDa


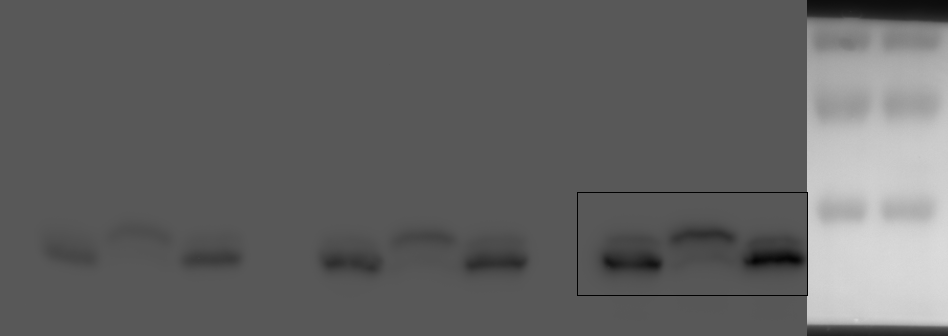


Anti-HA

100

70

55

Fig. 4c

kDa


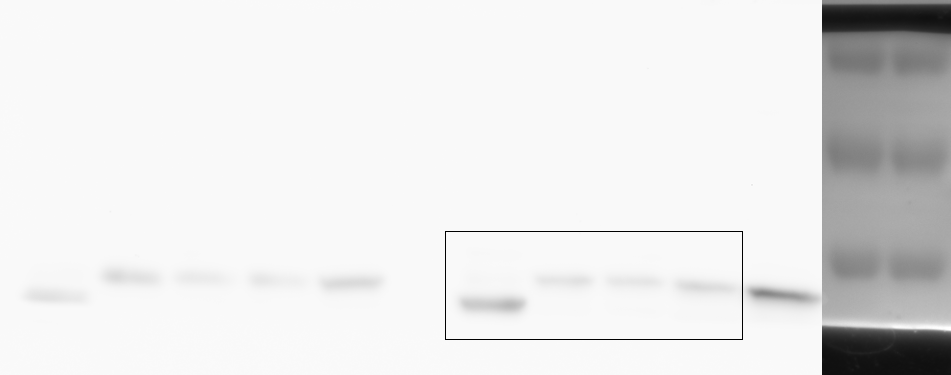


Anti-tagRFP

100

70

55

Fig. 5f


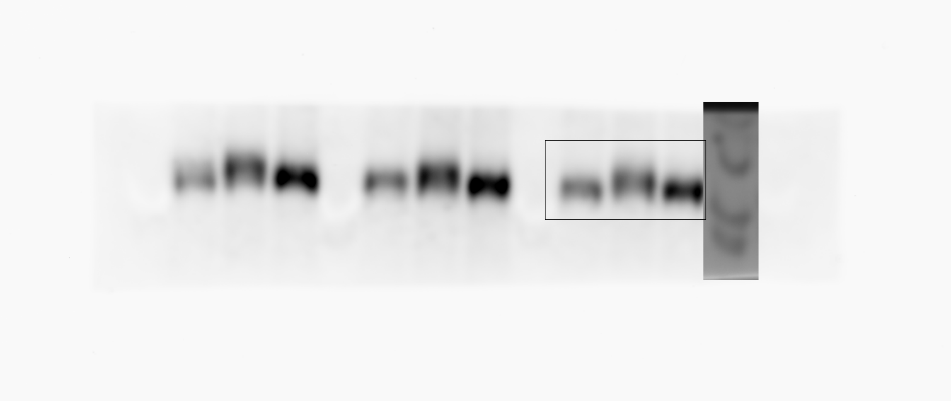


Anti-GFP

kDa

55

70

100

Fig. 5g


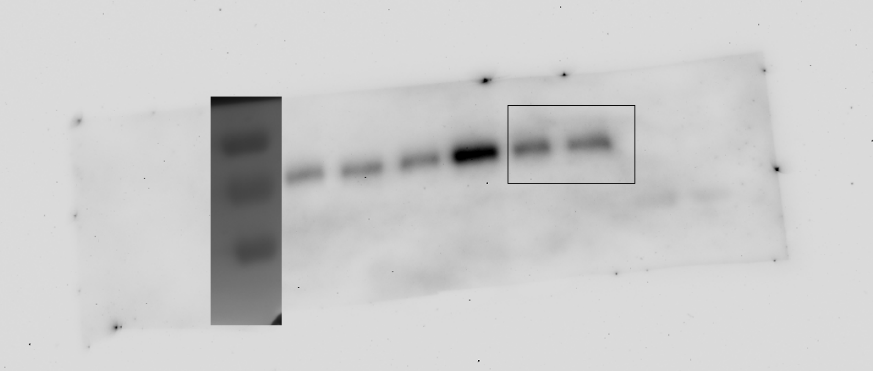


Anti-GFP

55

70

100

kDa

Fig. 5j


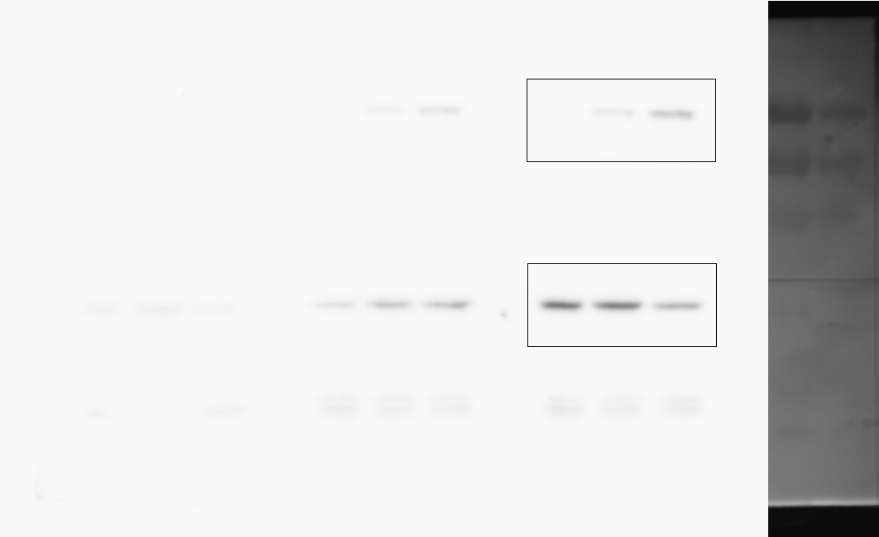


Anti-Por1

Anti-tagRFP

40

35

55

70

kDa

100

Fig. 6c

Pxp2-RFP

Pxp2-RFP-PTS

kDa

kDa


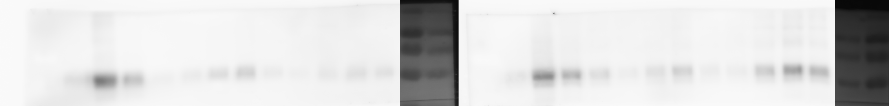


Anti-tagRFP

55

70

100

100

70

55


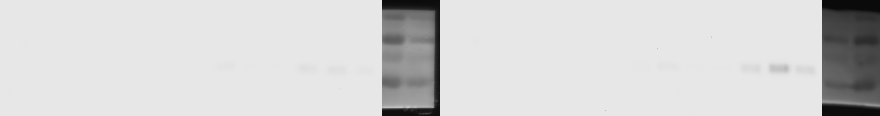


kDa

55

70

100

100

70

55

kDa

Anti-GFP


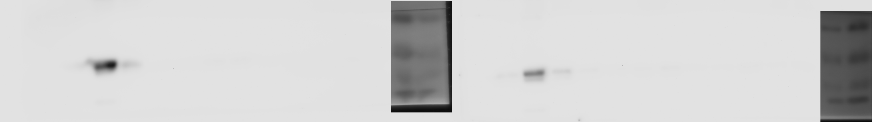


Anti-Por1

40

35

kDa

10

kDa

40

25

10

25

35

Fig. S2a

kDa

WT


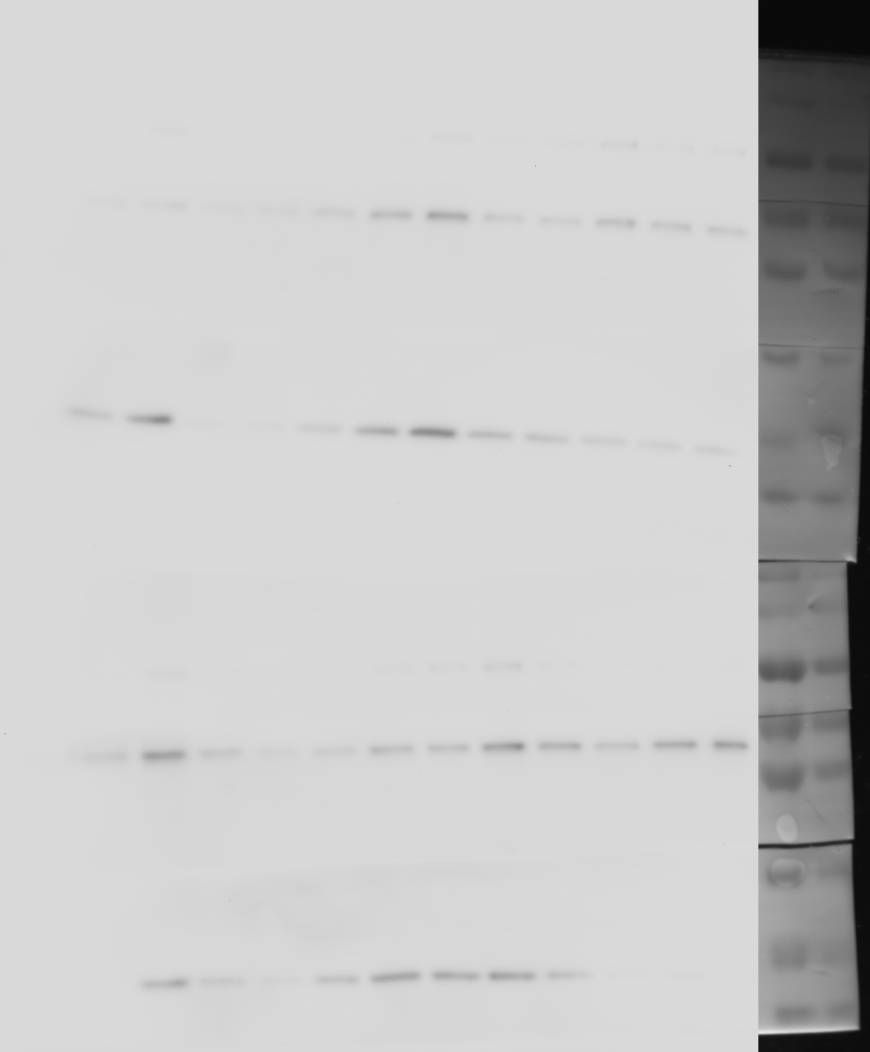


Anti-tagRFP

Anti-tagRFP

Anti-Por1

40

55

70

100

35

25

130

170

40

35

25

170

130

100

70

55

*Δimp1*

Anti-GFP

Anti-Por1

Anti-GFP

Fig. S2b

kDa

25


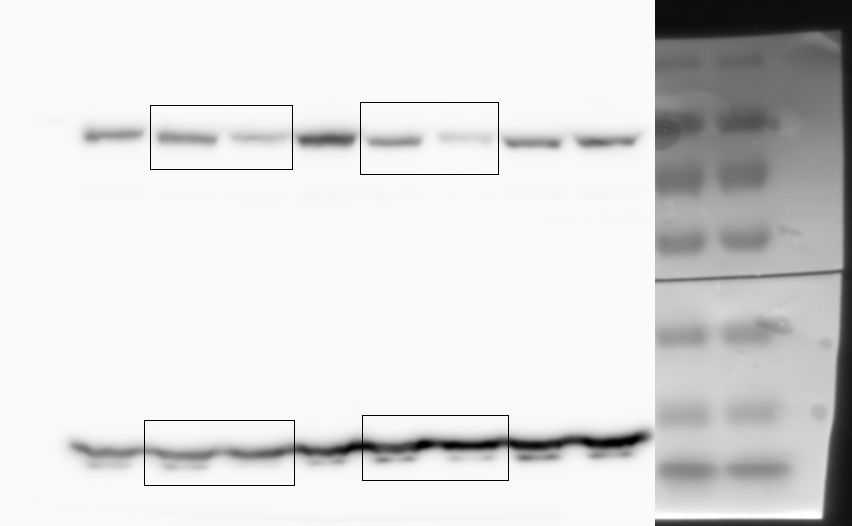


Anti-tagRFP

Anti-Por1

35

40

55

70

100

130

Ptc5-RFP

Ptc5-RFP-PTS

Fig. 2d (Blots used for quantification)

kDa


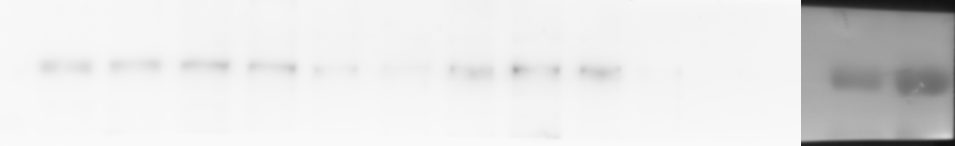


Anti-tagRFP

100


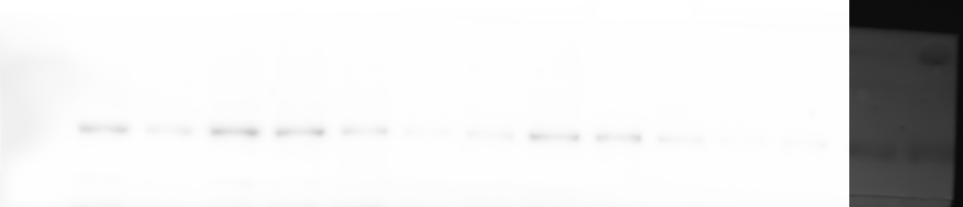


Anti-tagRFP

100

130

170


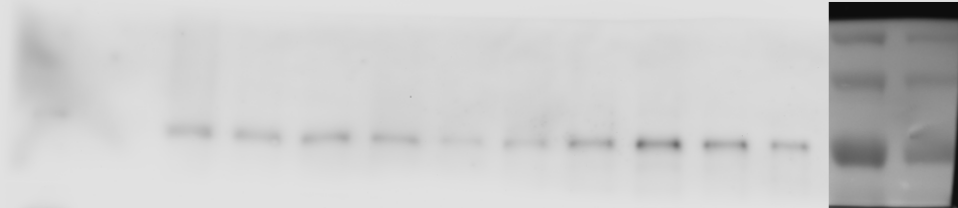


Anti-tagRFP

kDa

130

170

100


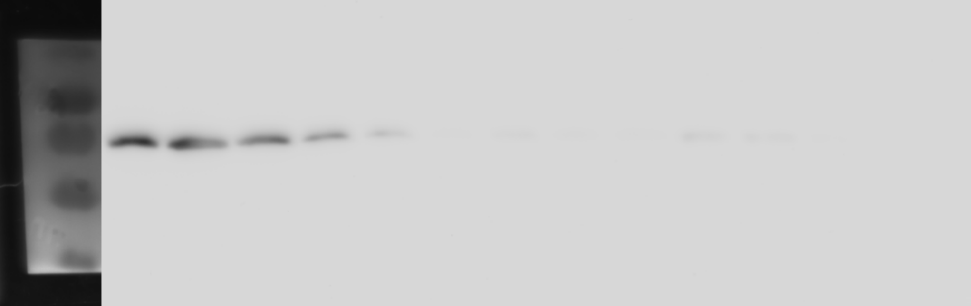


kDa

130

70

55

40

100

Anti-Myc


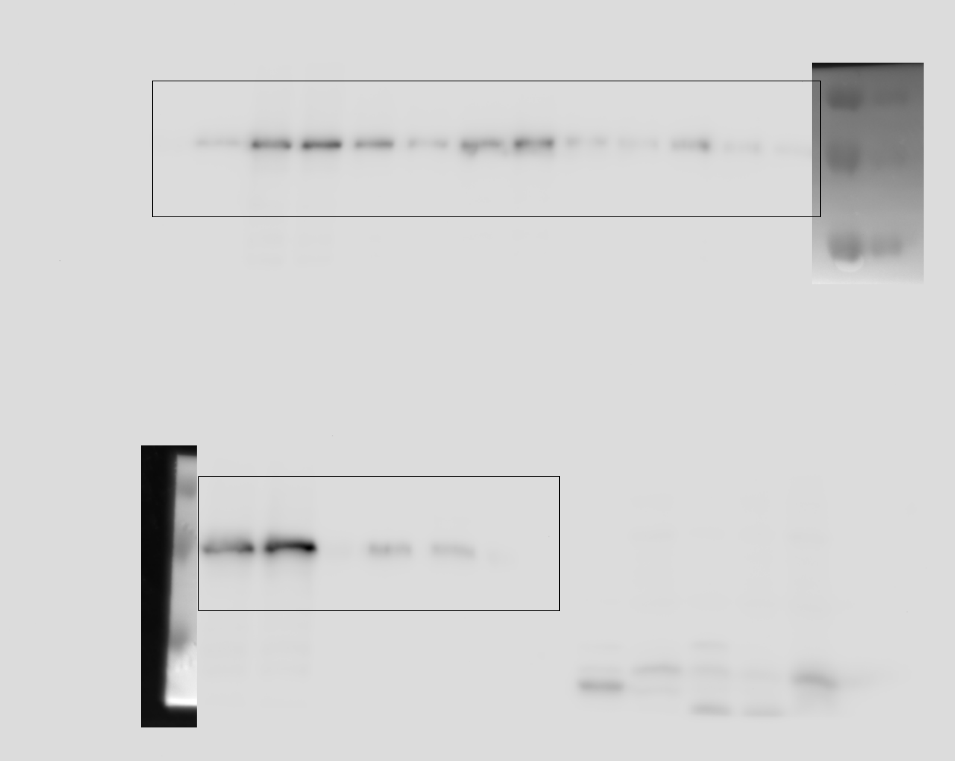


Anti-Myc

Anti-Myc

Ptc5-3xMyc-PTS; fractions 2, 3, 10 and 11

kDa

100

70

55

70

100

55

Fig. S3


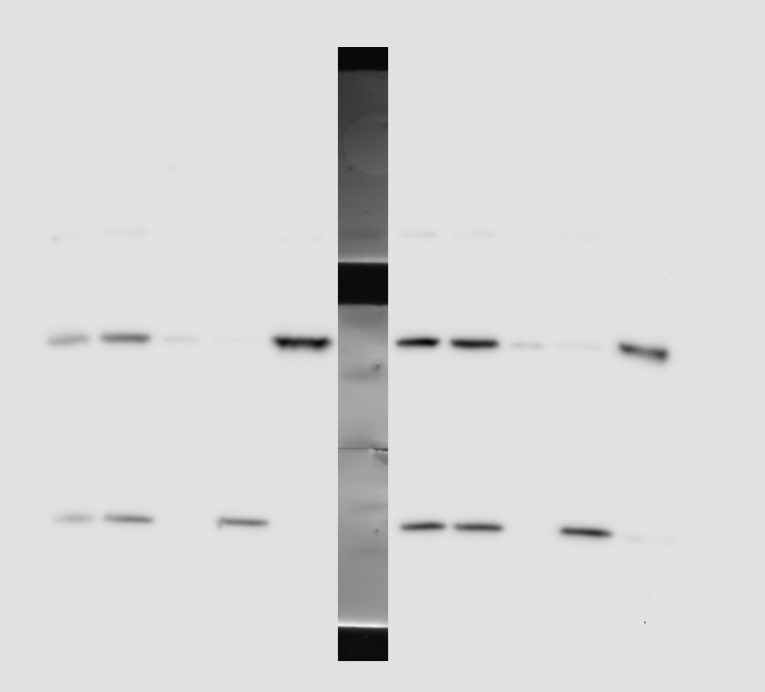


*Δimp1*

WT

Anti-tagRFP

Anti-Por1

Anti-GFP

kDa

25

35

40

55

70

100

130


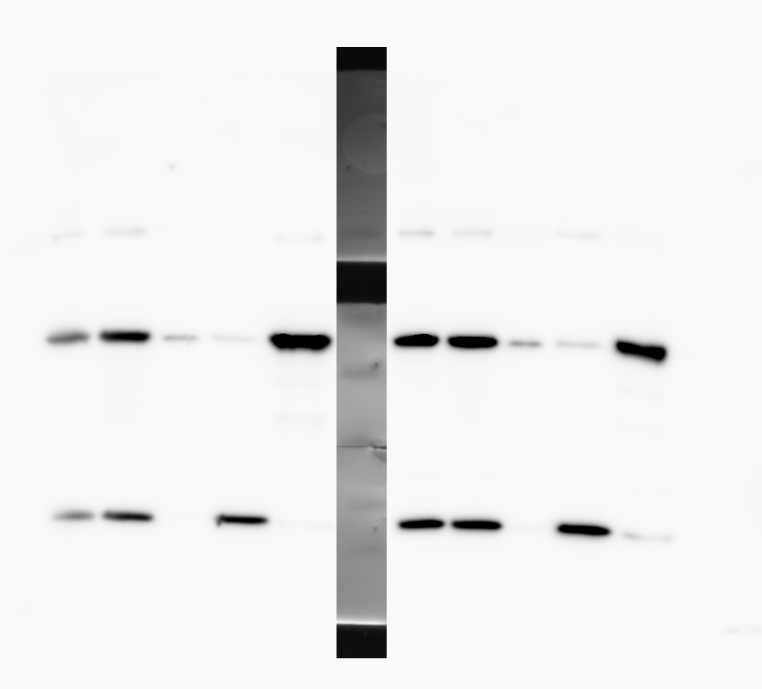


Longer exposure

100

70

55

40

35

25

kDa

130

Fig. S4a

kDa


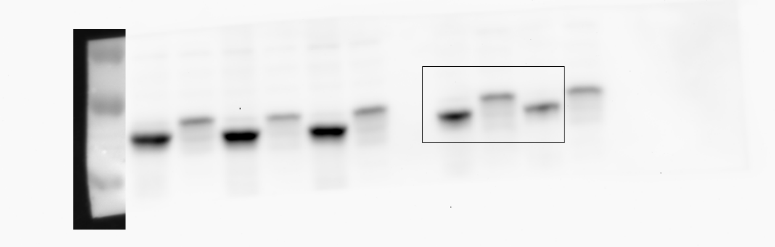


70

40

55

Anti-HA

Fig. S4c

kDa


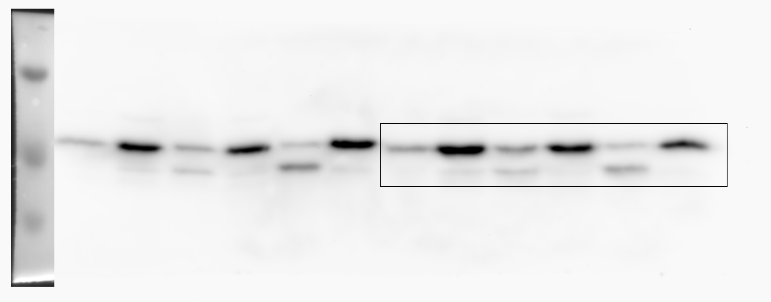


35

55

40

Anti-HA

Fig. S4d


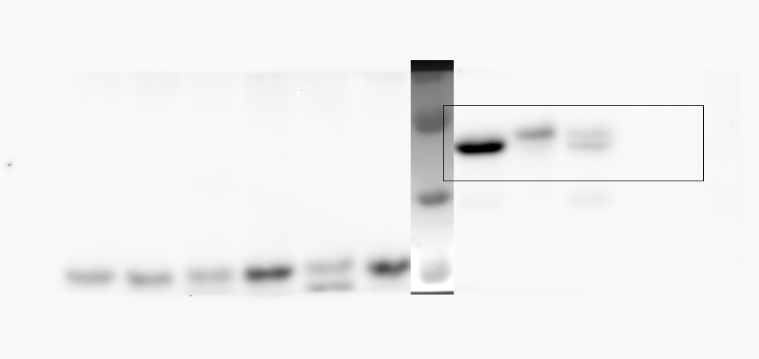


kDa

35

55

40

Anti-HA

Fig. S4e

kDa


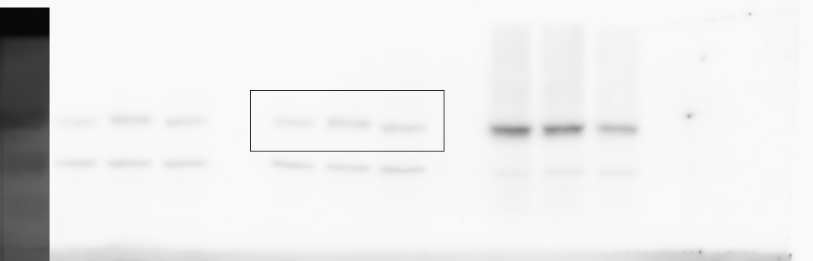


55

70

100

Anti-tRFP


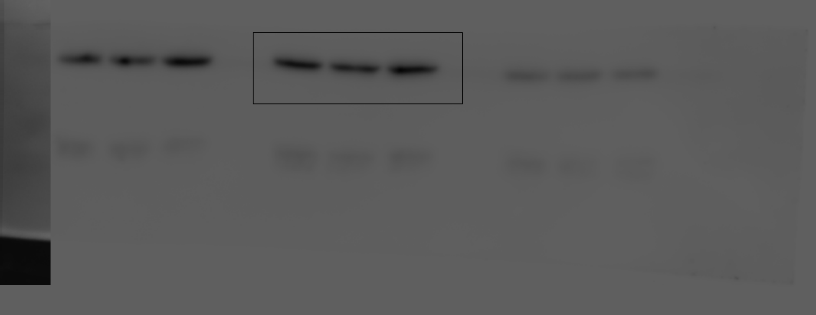


25

35

Anti- Por1

Fig. S6a

kDa

170

kDa

WT

*Δptc5*


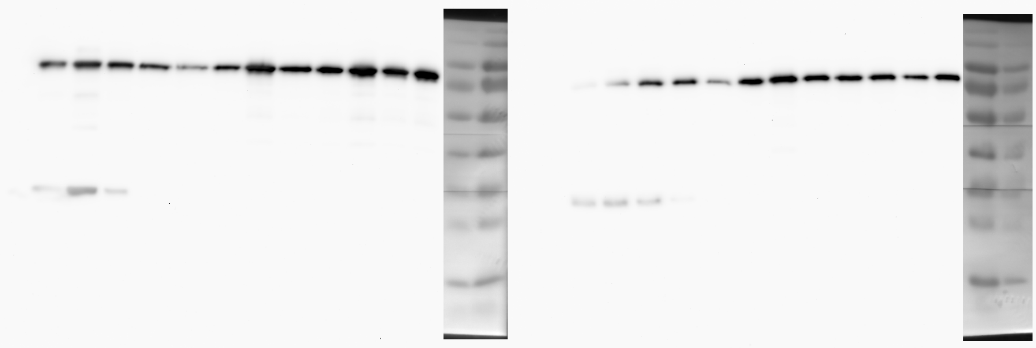


25

35

40

55

70

100

130

170

25

35

40

55

70

100

130

Anti-Por1

Anti-GFP
